# Deuterium isotope probing (DIP) on *Listeria innocua*: Optimisation of labelling and impact on viability state

**DOI:** 10.1371/journal.pone.0280885

**Published:** 2023-03-09

**Authors:** Sylvain Trigueros, Thomas Brauge, Tommy Dedole, Sabine Debuiche, Véronique Rebuffel, Sophie Morales, Pierre R. Marcoux, Graziella Midelet

**Affiliations:** 1 ANSES, Laboratory for Food Safety, Boulogne-Sur-Mer, France; 2 Department of Microtechnologies for Biology and Health, LETI, CEA, University Grenoble Alpes, Grenoble, France; University of Porto, PORTUGAL

## Abstract

An innovative approach, Raman microspectroscopy coupled with deuterium isotope probing (Raman-DIP), can be used to evaluate the metabolism of deuterated carbon source in bacteria and also to presume different anabolic pathways. This method requires the treatment of cells with heavy water that could affect the bacterial viability state at higher concentration. In this study, we evaluated the effect of heavy water incorporation on the viability state of *Listeria innocua* cells. We exposed the *L*. *innocua* suspensions to different heavy water concentrations (0%, 25%, 50% and 75%) from 30 minutes to 72 h of incubation times at 37°C. The total, viable and viable culturable populations were quantified by qPCR, PMA-qPCR and plate count agar respectively. We analyzed heavy water incorporation by Raman-DIP. The exposure of *L*. *innocua* cells to different concentrations of heavy water did not alter their cell viability to 24 h incubation time. In addition, the maximum intensity for C-D band, specific for the incorporation of heavy water, was reached after 2 h of exposure in a media containing 75% v/v D_2_O but an early detection of the labelling was possible at t = 1 h 30 min. In conclusion, the use of D_2_O as a metabolic marker was validated and can be developed for the detection of *L*. *innocua* cell viability state.

## Introduction

The bacteria of the *Listeria* genus are Gram-positive, facultative anaerobic and non-spore-forming. The genus *Listeria* consists of twenty-six closely related species with the addition of a new species, *Listeria swaminathanii*, in December of 2021 [1]. Of these species, only *Listeria monocytogenes* is a serious threat to humans, causing a lethal disease called listeriosis. The European Food Safety Authority report on zoonoses and food-borne illnesses in Europe in 2021 indicates that listeriosis ranks 5^th^ in terms of number of cases (with 4 to 5 cases per million inhabitants in Europe) but 1^st^ in terms of number of deaths, with an estimated mortality rate at 17.6% in 2019 [2]. This pathogenic bacterium, *L*. *monocytogenes*, represents a major concern for food safety, as it can also persist in the industrial environment and (re)contaminate food. *L*. *monocytogenes* has already been shown in several epidemics, such as in 2015, when a strain persisting in the industrial environment regularly contaminated salmon products, causing listeriosis cases in Denmark, Germany and France [3]. A few very sporadic infectious cases have been described for *Listeria innocua* [4, 5]. However, in view of the exceptional nature of these cases, *L*. *innocua* is considered non-pathogenic (class 1 bacteria) and remains the preferred non-pathogenic model in laboratory instead of *L*. *monocytogenes* (class 2 bacteria) in view of their biochemical similarities [6]. *L*. *monocytogenes* is able to attach and form biofilms on different materials type found in the food-processing environment [7] and has been isolated in the same niches as *L*. *innocua* [8]. Microbiological criteria (commission regulation EC No 2073/2005, article 5.2) require manufacturers in the agri-food sector to control batches of product intended for consumption as well as their environment (premises, installations, equipment). This makes it possible to control and detect the persistence of a certain strain in an industrial environment. This persistence may be due to its resistance to cleaning and disinfection procedures, which have been shown to induce a viable but non-culturable (VBNC) state in bacteria [9]. VBNC bacteria have very low metabolic activity and do not divide [10]. Therefore, VBNC bacteria do not grow on standard microbiological media, used during microbiological controls in industry [11], but retain the ability to become culturable again under favourable conditions. VBNC pathogens, if retain the ability to grow again, pose a risk to consumers in the food industry [12]. Microbiological standard techniques are long, not very specific and need complementary techniques to confirm the identification. Therefore, there is a need for rapid, reliable, and early detection of *L*. *innocua* and *L*. *monocytogenes* regardless of its viability state. To date, many rapid detection methods have been developed for *L*. *innocua* and *L*. *monocytogenes*, and the most commonly used are polymerase chain reaction (PCR) based methods [13]. These techniques provide reliable detection of the viability state of *L*. *innocua* [14] and *L*. *monocytogenes* [9], but have high quantification thresholds. In recent years, vibrational spectroscopies such as Raman are attracting a growing interest as they analyse the chemical composition of bacterial cells. Raman spectroscopy has the advantages to be fast, applicable to single-cell, label-free and non-destructive, and can give the identification down to species, like *Bacillus* species [15, 16]. This technique, coupled with Deuterium Isotope Probing (DIP), appears to be an innovative tool to probe metabolic activity in a variety of microbial genus (*E*. *coli*, *Bacillus subtilis*, *Bacillus thuringiensis*) [17]. The authors showed differences in cellular D uptake that were due to different anabolic pathways of glucose and naphtalene [18]. Nevertheless, this method requires the incorporation of heavy water (D_2_O) by the bacterial cell. It has been shown to be able to disrupt metabolic pathways in many organisms [19], disrupting growth and biofilm formation in *Pseudomonas aeruginosa* and *Streptococcus mutans* [20] with exposure to 75 or 100% of D_2_O potentially stressing the bacteria and altering its viability status. To our knowledge, no study has investigated the effect of this metabolic marker (D_2_O) on the viability state of *L*. *innocua* cells. In this study, we evaluated the impact of D_2_O incorporation on the viability state of *L*. *innocua* cells (Viable Culturable (VC), VBNC, dead) by classical microbiology and molecular biology methods. We validated D_2_O incorporation by Raman microspectroscopy.

## Material and methods

### Bacterial strain and bacterial suspension

A strain of *L*. *innocua* ATCC 33090 stored at -80°C in a heart-brain broth (Biokar Diagnostics, Beauvais, France) was plated on Trypticase Soy Agar with 0.6% Yeast Extract (TSAYE) (Oxoid, Basingstoke, United Kingdom) and incubated for 24 h at 37°C. Several colonies were then picked with a loop, and suspended in sterilized physiological water (double distilled water with 9 g/L of NaCl). Concentrations of bacterial suspension were adjusted to obtain a final concentration at 1.10^8^ CFU/mL.

### Preparation of deuterated and non-deuterated nutrient media

A Trypticase Soy Broth supplemented with 0.6% (w/w) Yeast Extract (TSBYE) (Biokar Diagnostics) was prepared with distilled water (non-deuterated) or with D_2_O at 99.9% atom (Sigma Aldrich, Saint Quentin Fallavier, France) to obtain different deuterated nutrient media of TSBYE with 25%, 50%, 75% (v/v) of D_2_O.

### Deuterated and non-deuterated bacterial suspension

One hundred microliters of the bacteria suspension at 1.10^8^ CFU/mL was centrifuged at 5000 g for 10 minutes and the supernatants were removed. The pellet was resuspended either in 1 mL of non-deuterated (TSBYE broth without D_2_O) or in 1mL of deuterated nutrient media (TSBYE broth with 25%, 50% or 75% v/v D_2_O). The tubes of non-deuterated and deuterated bacterial suspension were then placed at 37°C and incubated at different times: 30 minutes, 1 h, 1 h 30 minutes, 2 h, 4 h, 6 h, 24 h, 48 h or 72 h. After incubation, the tubes were centrifuged at 5000 g for 5 minutes, then the supernatants were removed and 1 mL of sterilized water were added. The cells were resuspended by vortexing. The procedure was repeated twice and the pellets were resuspended either in 1300 μL of sterilized water for microbiological and molecular biological analysis, or in 200 μL of sterilized water for Raman microspectroscopy. The sample prepared for microbiological analysis and molecular biological analysis was divided into three tubes: 200 μL for agar enumeration, 495 μL for propidium-monoazide (PMA)-qPCR, and 495 μL for qPCR analysis.

### Viable culturable bacterial enumeration

The suspensions were diluted to 10^−2^ and 50 μL were plated on TSAYE by a spiral plater (Easyspiral, Interscience, Saint Nom la Brétèche, France). After 24 h of incubation at 37°C, the bacteria were enumerated with a colony counter (Scan500, Interscience).

### Propidium monoazide treatment

Five microliters of 5 mM propidium monoazide (PMA) (Biotium, Fremont, USA) was added to 495 μL sample for PMA-qPCR, for a final concentration of 50 μM. Samples with PMA were incubated for 5 minutes at room temperature in the dark, then photoactived with 100% light exposure for 10 minutes in an Eppendorf tube using a PhAST Blue lamp photoactivation system (GenIUL, Terrassa, Spain).

### DNA extraction

The tubes without treatment or treated with PMA were centrifuged at 5000 g for 10 minutes at room temperature then the supernatant was removed. The pellet was resuspended in 180 μL of a lysis buffer, made with Tris(hydroxymethyl)aminomethane hydrochloride at 20 mM (Tris-HCl, Sigma Aldrich), Ethylenediaminetetraacetic acid (EDTA, Sigma Aldrich) at 2 mM; Triton X-100 (Sigma Aldrich) at 1.2% and lysozyme (Roche, Meylan, France) at 20 mg/ml. The DNA of samples without treatment or treated with PMA was extracted following the “Purification of Total DNA from Animal Tissues” of DNeasy®Blood & Tissue kit (Qiagen, Hilden, Germany). The elution step was carried out with 100 μL of AE buffer. The extracted DNA was then stored at -20°C.

### Total and viable bacterial quantification by qPCR

qPCR was performed with a LightCycler® 480 System (Roche) using TaqMan probe. The qPCR consisted of 10 μL of 2× QuantiTect Prob PCR mastermix (Qiagen), 1 μL of each primer (Eurogentec, Liège, Belgium), 0.3 μL of probe mix (Eurogentec), 5 μL of DNA template, and nuclease free water (MediaTech Inc, Manassas, USA) in a total volume of 20 μL. *L*. *innocua* specific primers and probe targeting the single copy *lin2483* gene [14]. Forward and reverse primers were as follows; 5′-ACATCAAGAAGAAGAAAAGCCTTATT-3′ (forward) and 5′-GCGTCGTTCCCATTCCATT-3′ (reverse). The probe sequence was 5′-FAM-CTTCCAATTCTTCTCGTTCTTGTCGTGC-BHQ-3′ which hybridized starting at nt +418 relative to the *lin2483* translation start site (+1). Thermocycling was performed at the following conditions: 10 minutes at 95°C followed by 40 cycles of 10 s at 95°C and 60 s at 60°C. Standard curve was obtained using *L*. *innocua* ATCC 33090 DNA covering the range from 10^1^ to 10^9^ genome equivalents (GE) per mL. The equation of the standard curve was y = -3.3677x + 49.227, with aR^2^ = 0.9878 and an efficacity of 99%.

### Raman microspectroscopic system for the D_2_O incorporation analysis

To each tube containing the pellet of non-deuterated or deuterated bacterial suspension, 200 μL of sterilized MilliQ water was added. One microliter was taken and placed on a clean quartz slide. The slide was then placed in the Raman system. A detailed description can be found in the works of these authors [16]. Briefly, the system allows targeting of bacterial cells thanks to imaging modalitied, and the measurement of single-cell Raman spectra using a confocal arrangement. The beam of a 532 nm, 50 mW laser (Spectra Physics Excelsior 532-50-CDRH) is attenuated and focused by a microscope objective (×100, 0.8 NA, Olympus LMPLFLN) in order to provide a spot size of 1 μm in diameter at the sample. Raman back-scattered light from an individual bacterium is collected by the same objective, filtered from Rayleigh light by a notch filter (NF03-532E, Semrock, New York, USA), and focused into the entrance fiber of a dispersive spectrometer (Hyperflux U1-532, Tornado Spectral systems, Toronto, Canada). The spectrometer featured at −15°C TE-cooled CCD, and spectral resolution of 10 cm^-1^ over the band 500–3400 cm^-1^. For each analysis point, namely a given configuration in terms of incubation time and D_2_O concentration, at least 60 bacteria cells were targeted and the corresponding Raman spectra were acquired. The Raman spectroscope acquisition parameter for each spectrum was tuned to 25 seconds and 250 mW, with our system that is a Lab custom system, not optimized for later industrial purpose.

### Raman spectra processing for D_2_O incorporation quantification

For each single cell, the acquired Raman spectra is a 2048-components vector sampling the 500–3500 cm^-1^ region. Each spectra is denoised using a Savitsky-Golay polynomial fit. Then a baseline removal is performed, using a Clayton algorithm. It is important to note that the regions of interest of the spectra for bacteria analysis, characteristic of species for instance, are approximatively 800–1800 cm^-1^ and 2800–3200 cm^-1^. The molecular bond C-D, due to the replacement of H by D in the CH bond, induces a peak at 2150 cm^-1^, in a spectra region showing no other information, thus independent of bacteria species. The peak is large, not too sensitive to noise, and occurs at a known location. Its height is estimated by comparing its absolute height level to the one of its neighbouring areas. This relative and local height estimation allows an implicit normalization, and the result is unitless. The ratio of the average of the C-D band to the average of the surrounding regions (CDN) was calculated using this method:

$$CDN=\frac{\frac{1}{K_{CD}}\sum_{k\in CD}Y_{k}}{\frac{1}{2}(\frac{1}{K_{{\mathrm{flat}}_{1}}}\sum_{k\in {\mathrm{flat}}_{1}}Y_{k}+\frac{1}{K_{{\mathrm{flat}}_{2}}}\sum_{k\in {\mathrm{flat}}_{2}}Y_{k})}$$

Where CD is equal to the integral of the C-D band from 2100 to 2200 cm^-1^, Flat_1_ is equal to the integral of the C-D neighboring band from 1900 to 2020 cm^-1^, and Flat_2_ is equal to the integral of the neighboring band from 2350, 2450 cm^-1^. The mean and variance of this band height is computed over the (about) 60 samples of the analyzed configuration to determine theimpact of D_2_O concentration and time of exposure of bacterial suspension.

### Statistical analysis

For molecular and microbiological analyses, all experiments were replicated six times (two replicates per experiment for three independent experiments). Data were analyzed using general linear model procedures in Statgraphics centurion V18 software (FRANCESTAT, Neuilly sur Seine, France). For all samples, analyses of variance (ANOVA) were performed to determine the impact of a) D_2_O concentration on bacterial suspension, b) time of exposure to non-deuterated and deuterated bacterial suspension on i) culturable, ii) viable, and iii) total populations of *L*. *innocua*. Differences were considered statistically significant at *p* < 0.05.

## Results

**Fig 1:**
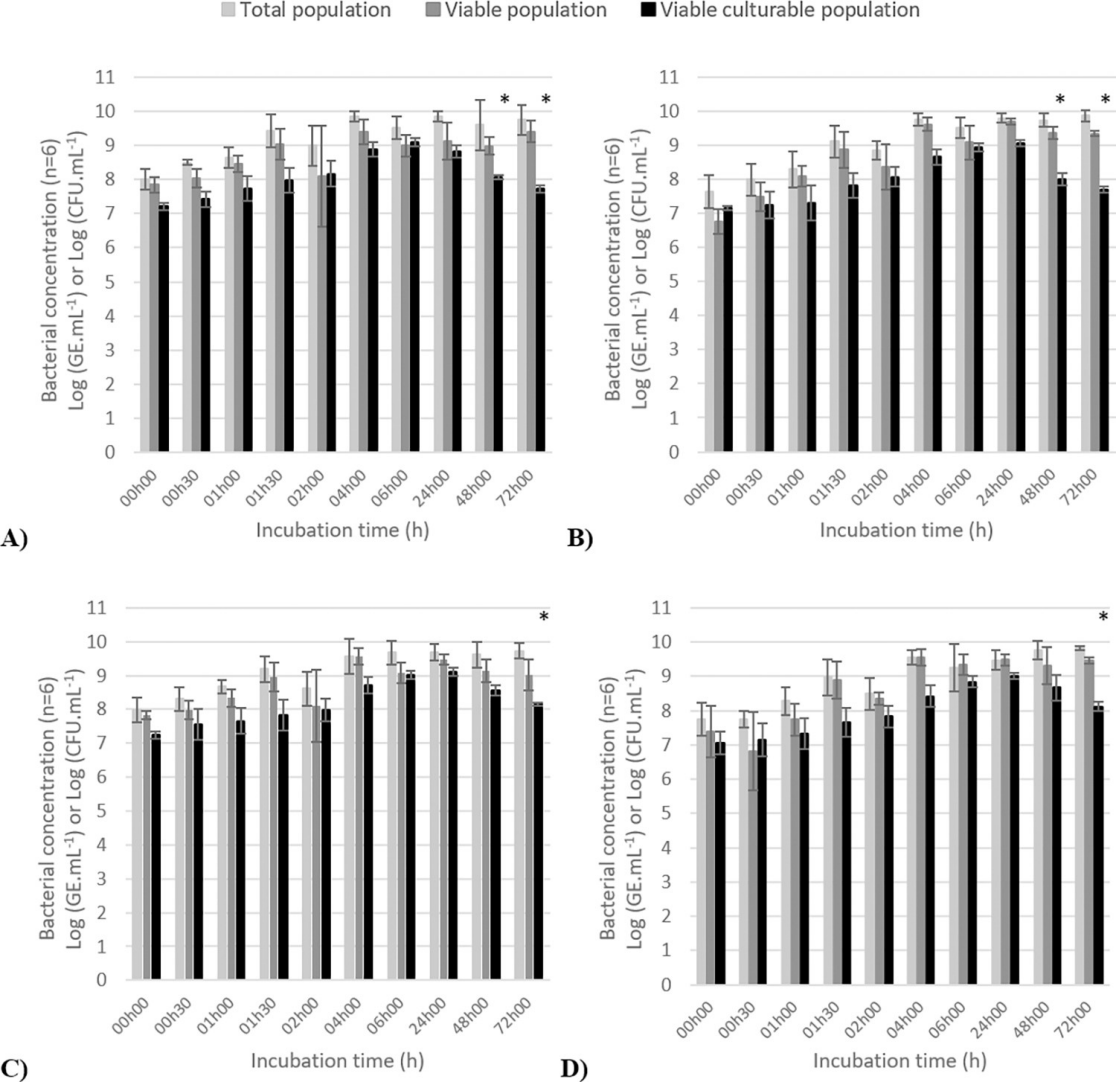
Quantification of total *L*. *innocua* ATCC 33090 population (Log (GE/mL)), viable population (Log (GE/mL)), and viable culturable population (Log (CFU/mL)) with A) 0%, B) 25%, C) 50% and D) 75% of D_2_O in nutrient media. The error bars represented the standard deviation (n = 6). Statistical significant differences were evaluated at *p*< 0.05 and represented by “*”.

Bacterial populations were quantified by plate counting (viable culturable population), PMA-qPCR (viable population) and qPCR (total population) after incubation of non-deuterated (control) and deuterated bacterial suspension (25%, 50% or 75% of D_2_O) at different times (Fig 1). The difference between quantification by qPCR assay and PMA-qPCR assay indicated the presence of a dead population and the difference between the quantification by PMA-qPCR assay and the enumeration on the agar plate indicated the presence of VBNC population. In the control experiment (non-deuterated bacterial suspension), the *L*. *innocua* populations increased from 8 to 9.7 log (GE/mL) for total population, and from 7.8 to 9.4 log (GE/mL) for viable population after 72 h of incubation at 37°C (Fig 1A). For the VC population, we observed an increase of this one from 7.2 log (CFU/mL) to 9.1 log (CFU/mL) after 24 h of incubation, and then a decrease to reach 7.7 log (CFU/mL) at 72 h. The viability state of *L*. *innocua* cells were VC for all incubation time, except at t = 48 h and at t = 72 h, demonstrating that a part of *Listeria* cells entered in a VBNC state. Similar tendencies have been observed for the quantification of VC, viable, and total populations in the three different deuterated (25%, 50%, 75%) bacterial suspensions until 24h exposure. Nevertheless, for the incubation time of 48 h, we observed the presence of VBNC population in the 25% deuterated bacterial suspension as well as in non-deuterated bacterial suspension (control), and at 72 h of incubation, we observed the presence of VBNC population in all deuterated and non-deuterated bacterial suspensions.

**Fig 2:**
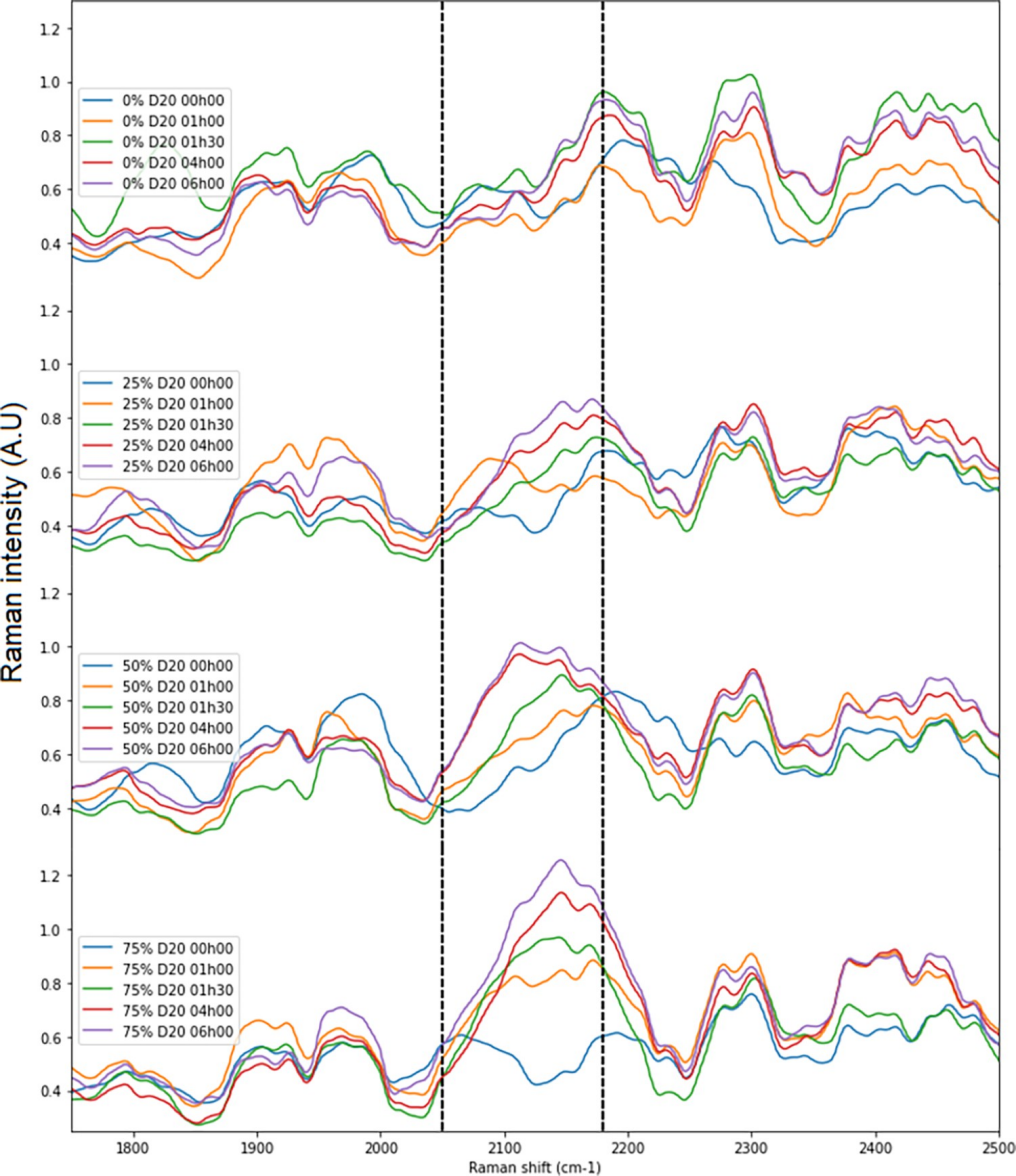
Raman spectra (1800–2500 cm^-1^) of single *L*. *innocua* cells grown in TSBYe with heavy water (0%, 25%, 50% and 75% D_2_O of nutrient medium water).

**Fig 3:**
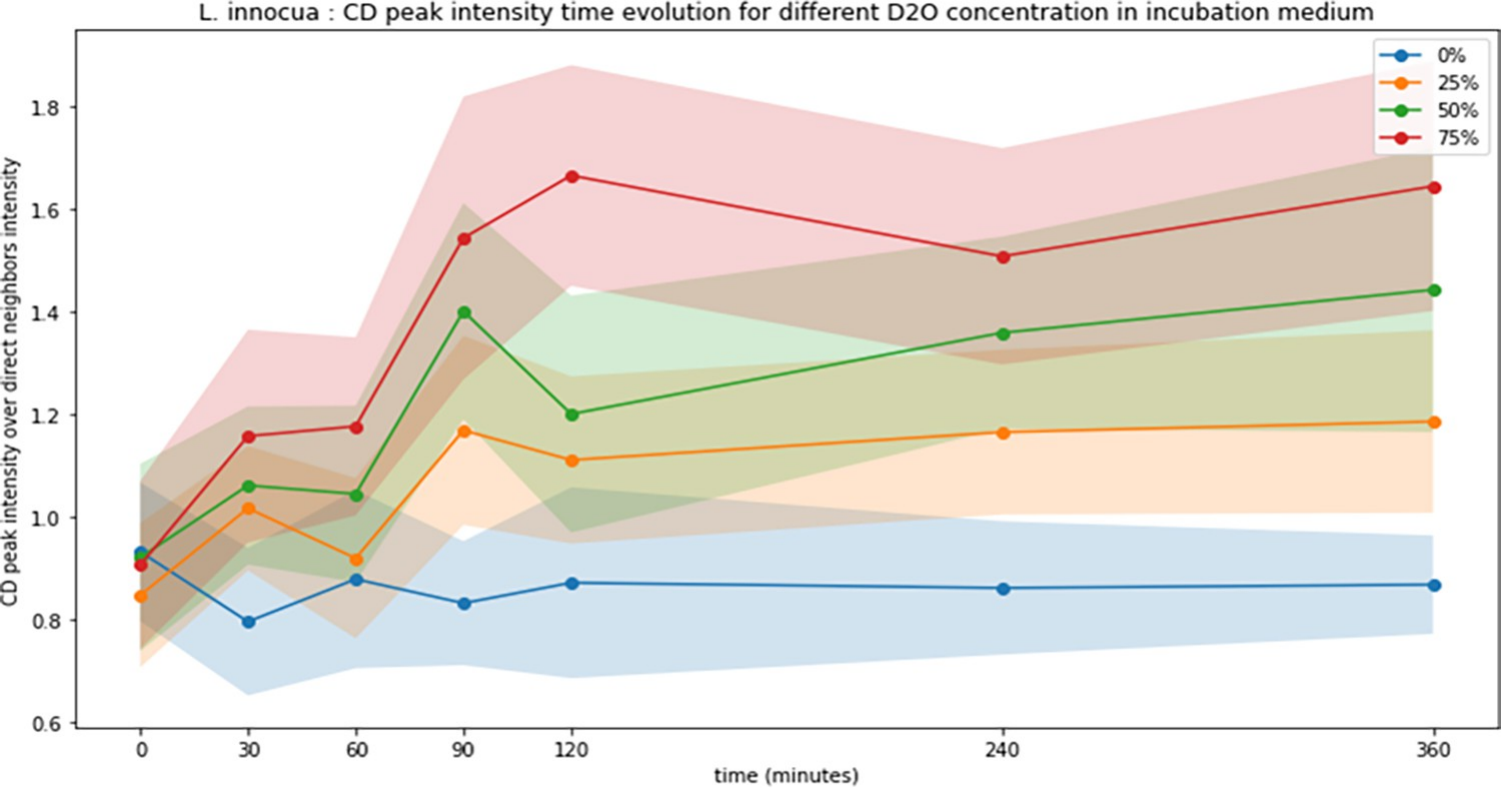
Quantification of the C-D band (between 2,040 and 2,300 cm^−1^) in non-deuterated (0% of D_2_O (curve blue) and deuterated (25% (curve orange), 50% (curve green), 75% (curve red) of D_2_O) bacterial suspension of *L*. *innocua* ATCC 33090. Each points represents the mean height of the C-D band (n > 60). The coloured halo represents one standard deviation (n > 60). Statistical significant differences were noticed when blue halo is separated to other colored halo.

For single-cell Raman spectra, we were interested in the effect of different incubation times and different D_2_O concentrations on the appearance and evolution of the C-D band (Fig 2; S1 Fig). The intensity of the C-D band after 6 h of incubation of *L*. *innocua* cells in nutrient medium containing 25% D_2_O was around 0.9 AU. This same intensity of the C-D band was reached after 1 h 30 of incubation in nutrient medium containing 50% D_2_O and after 1 h of incubation in nutrient medium containing 75% of D_2_O. The influence of incubation time on the C-D band height was also observed for each D_2_O concentration. We observed that for a given incubation time, if the D_2_O concentration increased, the intensity of the C-D band increased mainly in the 2050–2180 cm^-1^ area. Then, we calculated the ratio of the average of the C-D band to the average of the surrounding regions (CDN) for each incubation time and each D_2_O. We observed that the intensity values of the C-D band followed the same tendencies for all the different conditions with initially an increase in the height of the band from 2 h of incubation, then a stationary phase from 24 h (Fig 3). Nevertheless, the different concentrations tested give different C-D band heights. After t = 1 h 30 of incubation of deuterated bacterial suspension, the band height was of 0.175 with 25% D_2_O, 0.225 with 50% D_2_O and 0.3 with 75% D_2_O. By specifically observing the dynamics of the C-D band height for the three D_2_O concentrations, we observed that there was an optimum of deuterium labelling that was related to D_2_O concentration. The band height in 25% and 50% deuterated bacterial suspension reached a stationary phase for the t = 1 h 30 of incubation time. This same stationary phase was reached at t = 2 h for the deuterated bacterial suspension containing 75% D_2_O. At t = 4 h, the height of the C-D band decreased slightly, and then was up again t = 6 h. The condition to integrate the maximum of D_2_O in *L*. *innocua* was therefore t = 2 h of exposure in a 75% deuterated media, but an early detection of the labelling was possible at t = 1 h 30.

## Discussion

We were the first to evaluate the impact of labelling of D_2_O on the viability state of *L*. *innocua*. We studied the growth of *L*. *innocua* in the bacterial suspensions from 30 minutes to 72 hours in deuterated nutrient media (25% D_2_O, 50%, or 75%) at 37°C. The VC, viable and total bacterial populations were in the same quantities, of 7 log (CFU /mL) at t = 0 and increased to almost 9 log (CFU/mL) after 24 h of incubation time in all tested conditions. From this fact, only VC population was quantified in these bacterial suspensions and the VBNC or dead populations were not detected. Nevertheless, we detected a decrease of VC population, but the quantification of viable and total populations remained stable, suggesting the apparition of VBNC population after 48 h of incubation at 37°C in media without D_2_O and with 25% D_2_O, and at 72 h of incubation with 50% and 75% of D_2_O. The apparition of VBNC population in bacterial suspensions after 48 h or 72 h of incubation was not linked to incorporation of D_2_O by cells because it was observed also in the control experiment (without D_2_O). Incorporation of deuterium as a replacement for hydrogen did not cause sufficient stress to alter the viability state. However, it was possible that this was related to the depletion of the culture media. It has been shown in *L*. *monocytogenes* that starving bacteria can enter in VBNC state as a survival strategy in response to the nutrient stress [21, 22]. For the high D_2_O tested concentrations (50% and 75%), VBNC population appeared at 72 h of incubation while for control and 25% D_2_O concentration, VBNC population appeared earlier at 48 h of incubation. We suggest that high D_2_O concentrations may have slowed down the overall metabolism of the bacterial cells and thus delayed the depletion of the media in nutrients. At the moment, few works have showed an effect of D_2_O incorporation on bacterial cells because they were studied only the VC population. Indeed, in this study [17], bacterial cultures of *Escherichia coli*, *Bacillus thuringiensis*, and *Bacillus subtilis* containing different concentrations of D_2_O in nutrient medium at 37°C were followed at OD_578nm_ during 24 h. At high D_2_O concentration (100%), they observed a significant effect on reducing the growth rate of *E*. *coli* and *B*. *thuringiensis*, but not for *B*. *subtilis*. The authors have tested the effect of different concentrations of D_2_O on the temporal variation of OD_600nm_ for *Aeromonas* sp., *Pseudomonas* sp., *E*. *coli* and *Staphylococcus aureus* cultures [23]. They observed that only *Aeromonas* sp. showed slight growth inhibition for a concentration of 30% D_2_O or more. However, it was difficult to assess whether the D_2_O treatment had a stressful impact on these species because the OD of culture measured light scattering and not absorbance. The OD measured the evolution of dividing bacterial populations, the dead cells, the small air bubbles as living cells and the precipitation/ distort estimation of metabolic activity and it was directly related to the number of microorganisms in very low-density suspensions but a rather parabolic curve for higher density cultures. It was therefore not a sufficiently reliable measure to be able to assert the safety of D_2_O.

For the Raman measurements, the appearance of the C-D band in function of D_2_O concentration and incubation time was observed for *L*. *innocua* in bacterial suspension. The works reported the same type of observation on other bacterial species (*E*. *coli*, *B*. *thuringiensis*, *Bacillus subtilis*) [17]. The authors had observed that according to the type of metabolism of the cells, heterotrophic for *E*. *coli*, *B*. *thuringiensis* and *Bacillus subtilis*, autotrophic oxidizing nitrites for *Nitrospira moscoviensis*, autotrophic oxidizing ammonia for *Nitrosophaera gargensis* or autotrophic methanogens for *Methanobrevibacter smithii* and *Methanocorpusculum labreanum*, the appearance and intensity of the C-D band were different. Indeed, autotrophic microorganisms must reduce CO_2_ to produce biomass. This causes a stronger incorporation of hydrogen atoms from water than for heterotrophic organisms, and thus a stronger D incorporation. After 30 minutes of incubation, a part of the bacterial population in the deuterated media started to incorporate D_2_O with a presence of a C-D band on the spectra that was not observed on the control spectrum. After 2 h of incubation in deuterated media, a maximum incorporation level was reached, with a band height which did not vary over time but had a different maximum level depending on the concentration of D_2_O. A maximum intensity for C-D band, specific for the incorporation of heavy water, was reached after 2 h of exposure in a media containing 75% D_2_O but an early detection of the labelling was possible at t = 1 h 30. The replicates of measurements performed with the Raman spectroscope allowed us to have a statistical representation of the deuteration of the bacterial population, we saw that the level of deuteration of the cells (height of the C-D band) was positively correlated with the D_2_O concentration in the nutrient medium. These results were consistent with the works on *Bacillus* sp. and *E*. *coli* [17]. The authors have observed evolution of C-D band height in 30% of D_2_O, with an incubation time until 60 minutes for *Pseudomonas* spp., *E*. *coli*, *Aeromonas sp*. and *Staphylococcus aureus* [23]. They also showed that Gram negative bacteria (*Pseudomonas* spp., *E*. *coli*, *Aeromonas sp*.*)* had a higher C-D band than Gram positive bacteria (*Staphylococcus aureus*) with an equal exposure time to D_2_O, and this band increased with time. However, it is unclear whether the cultures, in that study, have reached a stationary phase in D incorporation because monitoring was stopped after 1 h of exposure. In our study, we have indeed observed that the amount of D incorporated by the cell was increasing until 1 h 30 at 25% and 50% of D_2_O and 2 h for 75% of D_2_O, followed by a stationary phase where the amount of D in the cell was stabilized. The undeniable advantage of Raman is the ability to characterize each cell individually in a sample and to give in a single measurement the information of the identification and the metabolic activity of the cell, which would be much faster and more precise than the cultivation methods currently used in companies. Moreover, the microscope coupled to the Raman spectrometer cannot visualize dead cells, which do not present any risk to human health.

## Conclusion

In this study, we determined the impact of the D_2_O incorporation on the viability state of *L*. *innocua* by following the viability state of the bacterial population over time and by measuring its incorporation level by Raman spectroscopy. We showed that D_2_O incorporation had no impact on the viability state of *L*. *innocua* cells for all concentrations studied. In addition, we optimized the labelling protocol for DIP and we observed that the maximum intensity for C-D band was reached, in *L*. *innocua*, after 2 h of exposure to a media containing 75% v/v D_2_O but an early detection of the labelling was possible at t = 1 h 30 minutes. In conclusion, the use of D_2_O as a metabolic marker was validated. Raman-DIP can be developed as a tool for the detection of metabolically activity of *L*. *innocua* cells in different viability state (VC, VBNC or dead). Raman spectroscopy is already use as an identification tool of stressed and non-stressed food-related bacteria [24]. DIP-Raman spectroscopy could have a very useful application in food processing plants to characterize metabolic activity of bacteria from sampling on food matrix or surfaces.

## Supporting information

S1 Data (XLSX)

S1 Fig Representative complete Raman spectra of single *L*. *innocua* cells grown in TSBYe with heavy water (0%, 25%, 50% and 75% D_2_O of nutrient medium water). (TIF)
