# Supplementary figures and images for: Deuterium isotope probing (DIP) on Listeria innocua: Optimisation of labelling and impact on viability state
Source: PLoS One. 2023 Mar 9;18(3):e0280885. doi: 10.1371/journal.pone.0280885 (PMC9997870; doi:10.1371/journal.pone.0280885)

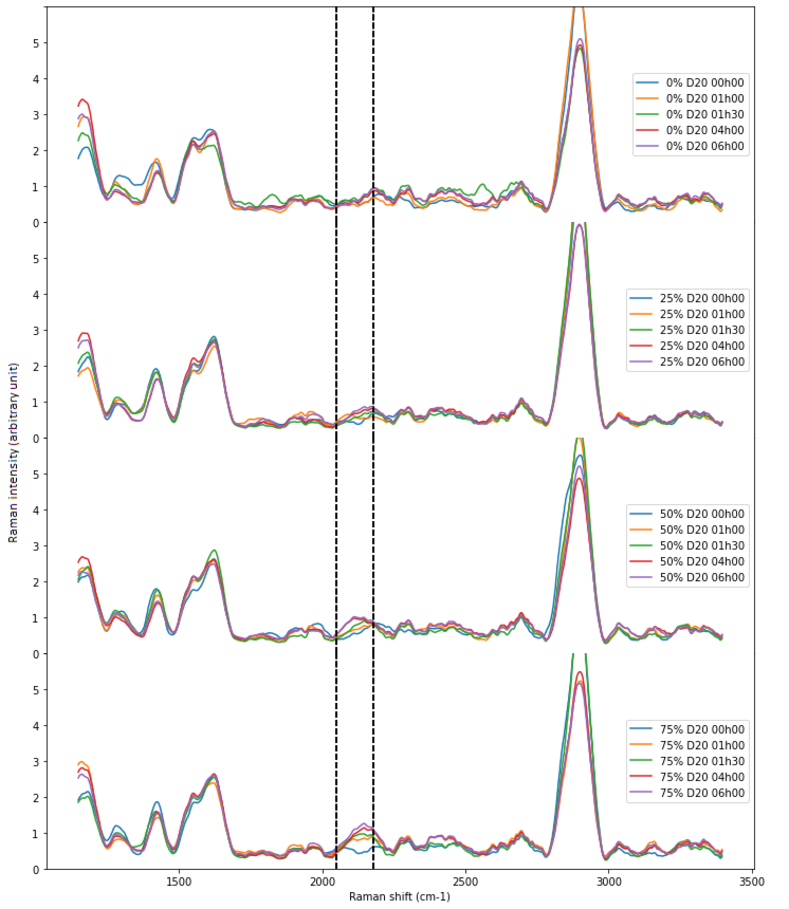

Supplement: S1 Fig — (TIF) [file pone.0280885.s002.tif]
